# The Development of Long-Term Adverse Health Effects in Oil Spill Cleanup Workers of the Deepwater Horizon Offshore Drilling Rig Disaster

**DOI:** 10.3389/fpubh.2018.00117

**Published:** 2018-04-26

**Authors:** Mark A. D’Andrea, G. Kesava Reddy

**Affiliations:** University Cancer and Diagnostic Centers, Houston, TX, United States

**Keywords:** blood disorders, cardiac toxicity, chemical exposure, crude oil spill, dispersants, health impact, hematological toxicity, hepatotoxicity, pulmonary toxicity

## Abstract

**Background:**

The purpose of this study was to assess the long-term adverse health effects of the 2010 *Deepwater Horizon* Gulf oil spill exposure in workers who participated in its cleanup work.

**Methods:**

Medical charts of both the oil spill exposed and unexposed subjects were reviewed. The changes in the white blood cells, platelets, hemoglobin, hematocrit, blood urea nitrogen, creatinine, alkaline phosphatase (ALP), aspartate amino transferase (AST), alanine amino transferase (ALT) levels, as well as their pulmonary and cardiac functions were evaluated.

**Results:**

Medical records from 88 subjects (oil spill cleanup workers, *n* = 44 and unexposed, *n* = 44) were reviewed during initial and 7 years follow up visits after the disaster occurred. Compared with the unexposed subjects, oil spill exposed subjects had significantly reduced platelet counts (×10^3^/µL) at their initial (254.1 ± 46.7 versus 289.7 ± 63.7, *P* = 0.000) and follow-up (242.9 ± 55.6 versus 278.4 ± 67.6, *P* = 0.000) visits compared with the unexposed subjects (254.6 ± 51.9 versus 289.7 ± 63.7, *P* = 0.008). The hemoglobin and hematocrit levels were increased significantly both at their initial and follow-up visits in the oil spill exposed subjects compared to the unexposed subjects. Similarly, the oil spill exposed subjects had significantly increased ALP, AST, and ALT levels at their initial and follow-up visits compared with those of the unexposed subjects. Illness symptoms that were reported during their initial visit still persisted at their 7-year follow-up visit. Notably, at their 7-year follow-up visit, most of the oil spill exposed subjects had also developed chronic rhinosinusitis and reactive airway dysfunction syndrome as new symptoms that were not reported during their initial visit. Additionally, more abnormalities in pulmonary and cardiac functions were also seen in the oil spill exposed subjects.

**Conclusion:**

This long-term follow-up study demonstrates that those people involved in the oil spill cleanup operations experiences persistent alterations or worsening of their hematological, hepatic, pulmonary, and cardiac functions. In addition, these subjects experienced prolonged or worsening illness symptoms even 7 years after their exposure to the oil spill.

## Introduction

**Figure 1:**
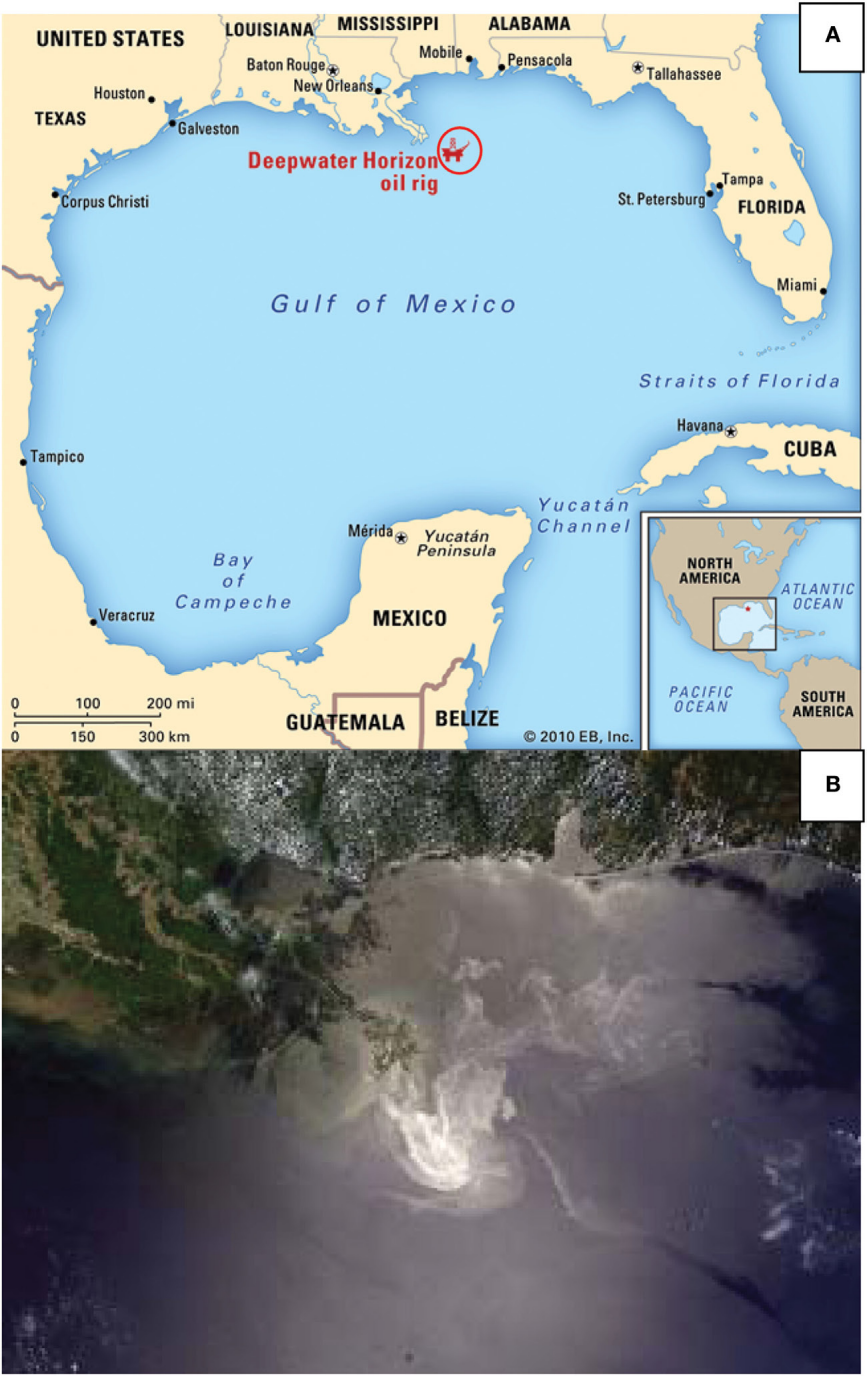
The *Deepwater Horizon* oil drilling rig. **(A)** The location of the *Deepwater Horizon* oil rig explosion in the Gulf of Mexico, 50 miles off the Louisiana coast (source: http://www.britannica.com/blogs/2011/04/deepwater-horizon-oil-spill-year-picture-essay-day/ by courtesy of Encyclopedia Britannica, Inc., Copyright 2010; used with permission). **(B)** Satellite view of the *Deepwater Horizon* oil spill slick by the United States National Aeronautics and Space Administration in the Gulf of Mexico [source: https://www.google.com/search?q=Satellite+view+of+The+Deepwater+Horizon+oil+spill+slick+by+the+United+States+National+Aeronautics+and+Space+Administration+in+the+Gulf+of+Mexico&source=lnms&tbm=isch&sa=X&ved=0ahUKEwiT9qXGmr3aAhUCY6wKHRkXAq4Q_AUICygC&biw=1366&bih=588#imgdii=MiZGQvd7BLCGrM:&imgrc=IaPOXnbdIqG6gM (Accessed: August 15, 2017)].

On April 20, 2010, an explosion of the British Petroleum (BP) operated offshore Deepwater Horizon drilling rig in the northern Gulf of Mexico resulted in one of the most catastrophic oil spill disasters in the history of the United States (Figure 1) (1). After several failed attempts to stop it, the well was finally capped 87 days later after over 4.9 million barrels of crude oil poured into the sea (2). As a consequence, more than 68,000 square miles area was contaminated affecting the coastal zone spanning from Texas to Florida through Louisiana, Mississippi, and The Alabama Gulf coast (Figure 1) (3–5). During the cleanup efforts, nearly two million gallons of dispersants such as Corexit (6) was released into the Gulf of Mexico to break up the crude oil slick (7). Tens of thousands of workers and volunteers responded to aid in the cleanup activities along this coastal zone (8). The magnitude of this oil spill has not only impacted the health of not only the cleanup workers but also those living in the affected area and also has implications for other oil spills that may occur around the world.

Crude oil is a complex mixture of a host of volatile organic chemicals including toxic chemicals such as benzene, toluene, xylene, ethylbenzene, and host of other chemicals. The Health Hazard Evaluation Report by the Centers for Disease Control and Prevention has concluded that cleanup responders had directly faced toxic chemical exposures from the BP oil spill disaster (9). Thus, the Gulf oil spill disaster has raised numerous questions about the immediate and long-term impact on the health of the cleanup workers, as well as those living in the surrounding oil-exposed regions and communities. The Gulf Long-term Follow-up (GuLF) STUDY had reported that exposure to low doses of benzene and other volatile organic compounds such as toluene, ethylbenzene, oxylene, xylene, and styrene is associated with adverse hematologic effects in study participants (10). The GuLF STUDY is currently conducting further research to examine oil spill-related adverse health effects utilizing biological specimens from study participants (11).

Despite the significant health risks, relatively little is understood about the adverse human health effects of toxic exposures from oil spill disasters. Notably, most of the earlier studies that reported in literature have focused primarily on the physical effects and psychological sequelae in oil spill affected populations (12–15). Recent review articles by the authors and other investigators revealed that very few studies have attempted to assess the psychological well-being and human health from more than 40 oil spill disasters that have occurred around the world (12, 13, 16). These reviewers have found that the majority of the studies reported a spike in acute physical and mental health symptoms following a oil spill exposure. These symptoms included headache, shortness of breath, fatigue, cough, skin rash, diarrhea, low back pain, depression, anxiety, and posttraumatic stress syndrome following the spill (17–23). Earlier studies have also shown that the cleanup workers and local residents had an increased prevalence of respiratory symptoms immediately after their exposure to the oil spill (8, 24, 25) and that these effects persisted for prolonged time periods after the spill (26–28). A recent longitudinal study on the psychological effects of the oil spill reported that women who were more highly exposed experienced higher levels of depressive symptoms and mental distress than women who were less exposed to the Deepwater Horizon oil spill (29).

Recently, we reported that workers involved in the cleanup activities of the Deepwater Horizon oil spill experienced significantly altered hematological and hepatic functions and had a higher prevalence of illness symptoms (13, 30, 31). Unfortunately, to date, there have been no longitudinal studies that have evaluated the long-term adverse health effects of the Gulf oil spill exposure in those participants who were involved in its cleanup activities. The purpose of this study is to address the paucity of longitudinal data on the adverse health effects of the oil spill exposure by assessing the changes in hematological and hepatic functions among those workers who were involved in oil spill cleanup operations. More specifically, we assessed the adverse health effects of the oil spill exposure on cleanup workers during their initial visit and 7 years later after the disaster occurred. In addition, we examined the prevalence of illness symptoms, pulmonary and cardiac functions in the oil spill cleanup workers during their initial visit and 7 years later after the disaster occurred.

## Materials and Methods

### Subjects

Ethical approval for this study protocol was obtained from an external accredited Institutional Review Board. This study was conducted according to the ethical principles of the Declaration of Helsinki. To comply with the Health Insurance Portability and Accountability Act, confidentiality of information was secured by utilizing text encryption, password protection, and limited personnel involvement in data collection.

The details of the subjects’ selection and the procedures employed for the clinical and laboratory evaluations have been reported previously (30, 31). Briefly, the subjects exposed to the oil spill were identified as participants in the oil spill cleanup activities along the Gulf coast. Using their medical charts, demographic and clinical data were reviewed. Subjects included in the study underwent clinical and laboratory evaluations by an experienced physician. The unexposed subjects selected for the study were living geographically at least 100 miles away from the Gulf coast where the oil spill occurred. The unexposed subjects had visited their family physicians office for a routine wellness checkup. The subjects were selected randomly by their primary care physicians for this study.

In our previous study (30), we assessed the adverse health effects of the oil spill exposure in 117 workers who were involved in its cleanup operations. In this follow-up study, only 44 of the 117 workers elected to return for further health evaluations 7 years after the oil spill disaster. The reasons for not returning for their follow-up health assessment are not completely known as many were not reachable due to changes in their contact information or they had moved out of the area.

### Chart Review and Data Gathering

Study investigators reviewed clinical data of the oil spill exposed and unexposed subjects. Laboratory data such as white blood cell (WBC) counts, platelet counts, hemoglobin, hematocrit, blood urea nitrogen (BUN), creatinine, alkaline phosphatase (ALP), aspartate amino transferase (AST), and alanine amino transferase (ALT) levels were collected and analyzed. Data of the initial and long-term illness symptoms, pulmonary and cardiac functions were also reviewed and evaluated.

### Statistics

Descriptive statistics were used to assess subjects’ demographics and included means and SDs for each group. Variables included were WBC, platelets, hemoglobin, hematocrit, creatinine, BUN, ALP, AST, and ALT. Student’s *t*-test was used to assess the differences between the oil spill exposed and the unexposed groups. The significance level was predetermined at an alpha level of 0.05.

## Results

We previously reported on the early health effects of the BP oil spill exposure in 117 individuals who participated in its cleanup activities. Of the 117 people intended for follow-up evaluations, only 44 elected to return for further health evaluations 7 years after the oil spill disaster. This study also included data from 44 unexposed subjects in the comparison group. The mean age of the unexposed subjects was 51.1 years. The mean age of the oil spill exposed subjects at their initial and later follow-up analysis was 38.2 and 43.1 years, respectively. There were six female subjects in each of the unexposed and oil spill exposed groups.

The findings in Table 1 represent the hematologic and hepatic profiles for the unexposed versus the exposed groups at their initial and follow-up visits. Although the oil spill cleanup workers initially had marginally increased mean WBC counts (×10^3^/µL) (7.2 ± 1.9 versus 6.5 ± 2.1, *P* = 0.058), no significant changes in WBC counts were found at their follow-up visit when compared with those of the unexposed group (6.7 ± 1.9 versus 6.5 ± 2.1, *P* = 0.474). Compared with the unexposed subjects, the oil spill exposed subjects had a significantly increased mean hemoglobin (g/dL) levels during their initial (14.9 ± 1.3 versus 13.6 ± 2.0, *P* = 0.000) and follow-up (14.9 ± 1.2 versus 13.6 ± 2.0, *P* = 0.001) visits. The hematocrit (%) levels were also significantly elevated in the oil spill exposed subjects at their initial (45.0 ± 3.1 versus 41.0 ± 5.3, *P* = 0.000) and follow-up visits when compared with the unexposed subjects (43.9 ± 2.9 versus 41.0 ± 5.3, *P* = 0.008). Conversely, the oil spill exposed subjects had significantly reduced platelet counts (×10^3^/µL) at both their initial (254.1 ± 46.7 versus 289.7 ± 63.7, *P* = 0.000) and follow-up (242.9 ± 55.6 versus 278.4 ± 67.6, *P* = 0.000) visits compared with the unexposed subjects (254.6 ± 51.9 versus 289.7 ± 63.7, *P* = 0.008). Similarly, the oil spill exposed subjects had significantly decreased BUN (mg/dL) levels at their initial (12.9 ± 3.3 versus 17.0 ± 4.9, *P* = 0.003) as well as their follow-up visits compared with the unexposed subjects (12.0 ± 2.8 versus 17.0 ± 4.9, *P* = 0.002). However, no significant differences were observed in the serum creatinine levels (mg/dL) among the oil spill exposed subjects either at their initial or follow-up visits compared with those of the unexposed subjects.

**Table 1:** Comparison of hematologic and hepatic indices between the unexposed and exposed subjects to oil spill (44 each group).

Variable	Unexposed	Exposed Visit 1	Exposed Follow-up	*P* value UE versus visit 1	*P* value UE versus F/U	*P* value Visit 1 versus F/U
WBC (×10^3^/μL)	6.5 ± 2.1	7.2 ± 1.9	6.7 ± 1.7	0.058^ψ^	0.474^ψ^	0.065^ψ^
Platelets (×10^3^/μL)	289.7 ± 63.7	254.1 ± 46.7	254.6 ± 51.9	0.002*	0.005*	0.485^ψ^
Hemoglobin (g/dL)	13.6 ± 2.0	14.9 ± 1.3	14.9 ± 1.2	0.000*	0.001*	0.457^ψ^
Hematocrit (%)	41.0 ± 5.3	45.0 ± 3.1	43.9 ± 2.9	0.000*	0.008*	0.080^ψ^
BUN (mg/dL)	17.0 ± 4.9	12.9 ± 3.3	12.0 ± 2.8	0.003*	0.002	0.119^ψ^
Creatinine (mg/dL)	1.0 ± 0.3	1.0 ± 0.2	1.0 ± 0.1	0.322^ψ^	0.407^ψ^	0.134^ψ^
ALP (IU/L)	63.5 ± 17.4	77.3 ± 10.6	72.4 ± 10.9	0.001*	0.022*	0.149^ψ^
AST (IU/L)	20.4 ± 5.3	33.1 ± 9.8	28.0 ± 10.0	0.004*	0.014*	0.199^ψ^
ALT (IU/L)	21.4 ± 6.5	36.7 ± 13.3	32.3 ± 12.0	0.000*	0.008*	0.214^ψ^

*P = 0.001.

*^ψ^Did not reach statistical significance*.

*WBCs, white blood cells; BUN, blood urea nitrogen; ALP, alkaline phosphatase; AST, aspartate amino transferase; ALT, alanine amino transferase*.

*UE, unexposed; F/U, follow up*.

Assessment of serum liver enzymes revealed that the oil spill exposed subjects had significantly elevated levels of ALP (IU/L) at their initial (77.3 ± 10.6 versus 63.5 ± 17.4, *P* = 0.001) and follow-up visits compared with those of the unexposed subjects (72.4 ± 10.9 versus 63.5 ± 17.4, *P* = 0.022). Similarly, the oil spill exposed subjects had significantly elevated levels of AST (IU/L) at their initial (33.1 ± 9.8 versus 20.4 ± 5.3, *P* = 0.004) as well as their follow-up visits compared with the unexposed subjects (28.0 ± 10.0 versus 20.4 ± 5.3, *P* = 0.014). Moreover, the levels of ALT (IU/L) were also significantly elevated in the oil spill exposed subjects at their initial (36.7 ± 13.3 versus 21.4 ± 6.5, *P* = 0.000) and follow-up visits when compared with the unexposed subjects (32.3 ± 12.0 versus 21.4 ± 6.5, *P* = 0.008).

To determine if there were any changes to the hematological and hepatic functions that occurred after exposure to the oil spill disaster, we compared the later follow-up clinical findings with those of the initial clinical findings of the oil spill exposed subjects. These findings indicated that there was no improvement in the altered hematological and hepatic functions between the earlier initial visit and the later follow-up visit indicating a prolonged and persistent adverse health effect due to the oil spill exposure even 7 years after the disaster (Table 1).

Several studies have previously shown that subjects exposed to an oil spill experienced multiple illness symptoms compromising their health. Therefore, we evaluated the incidence of illness symptoms and health complaints in the oil spill cleanup workers during their initial and subsequent follow-up visits. The results are presented in Table 2. Shortness of breath was the most frequently reported symptom among the oil spill exposed subjects at both their initial (75%) and at their 7-year follow-up (84%) visits. Headaches was the second most frequently reported symptom followed by skin rash, chronic cough, weakness, dizzy spells, painful joints, and chest pain experienced by the oil spill exposed subjects at both their initial and at their 7-year follow-up visits. The incidence of the most frequently occurring illness symptoms was comparable at both their initial and at their 7-year follow-up visits. However, at the 7-year follow-up visit, 91% of the oil spill exposed subjects had progressive deterioration of their respiratory system and developed chronic rhinosinusitis and 45% of the exposed workers had developed chronic reactive airway dysfunction syndrome as new symptoms that were not reported during their initial visit (Table 2).

**Table 2:** Incidence of somatic symptoms complaint by the oil spill cleanup works during initial visits and 5 years after the Gulf oil spill exposure.

Illness symptom	Initial visit (*N* = 44)	5-Year follow-up (*N* = 44)
Shortness of breath	33 (75%)	37 (84%)
Frequent headaches	28 (64)%	30 (68%)
Rash	23 (52%)	24 (55%)
Chronic cough	20 (45%)	21 (48%)
Weakness	19 (43%)	17 (39%)
Dizzy spells	15 (34%)	14 (32%)
Painful joints	13 (30%)	12 (27%)
Chest pain	12 (27%)	15 (34%)
Night sweats	12 (27%)	6 (14%)
Frequent diarrhea	10 (23%)	7 (16%)
Heartburn	10 (23%)	5 (11%)
Hoarseness	9 (20%)	9 (20%)
Numbness	9 (20%)	5 (11%)
Ringing in ears	9 (20%)	4 (9%)
Nasal obstruction	7 (16%)	10 (23%)
Poor appetite	7 (16%)	6 (14%)
Swelling of ankles	7 (16%)	6 (14%)
Double vision	6 (14%)	7 (16%)
Nosebleeds	6 (14%)	2 (5%)
Black tarry stools	5 (11%)	4 (9%)
Difficulty swallowing	5 (11%)	4 (9%)
Memory loss	5 (11%)	3 (7%)
Palpitations	4 (9%)	5 (11%)
Blindness	3 (7%)	2 (5%)
Loss of balance	3 (7%)	3 (7%)
Pain	3 (7%)	7 (16%)
Urinary frequency	3 (7%)	0 (0%)
Blood in urine/sputum	2 (5%)	2 (5%)
Constipation	2 (5%)	1 (2%)
Difficulty breathing	2 (5%)	4 (9%)
Discoordination	2 (5%)	1 (2%)
Hearing loss	2 (5%)	1 (2%)
Slow stream	2 (5%)	2 (5%)
Burning on urination	1 (2%)	0 (0%)
Dental problems	1 (2%)	1 (2%)
Painful urination	1 (2%)	0 (0%)
Skin lesions	1 (2%)	2 (5%)
Chronic rhinosinusitis	0 (0%)	40 (91%)
Reactive airway dysfunction syndrome	0 (0%)	20 (45%)

**Figure 2:**
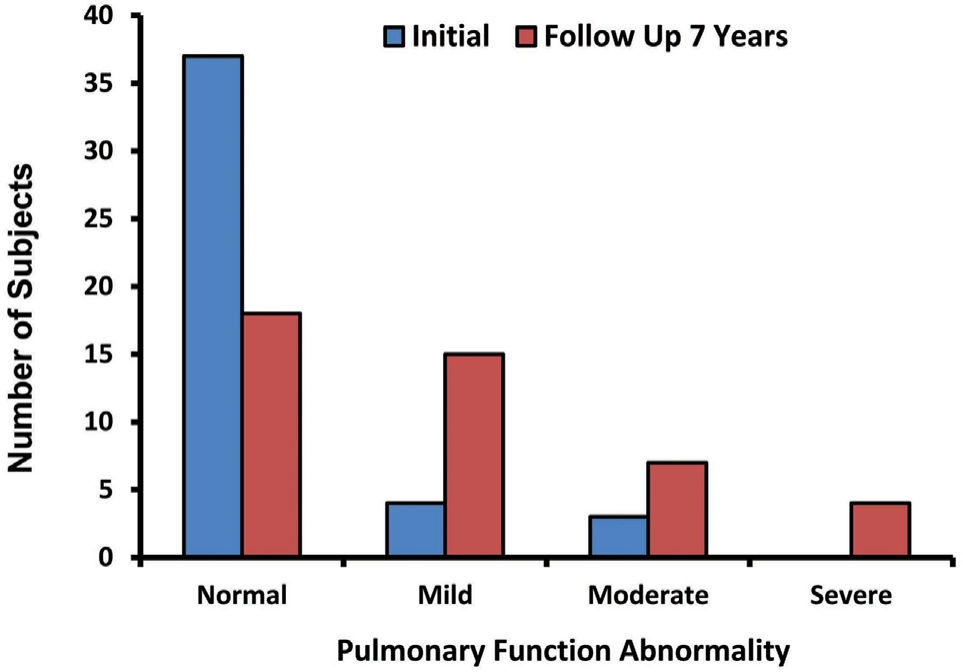
Pulmonary function abnormalities among the oil spill cleanup workers during their initial and later follow-up visits.

The abnormalities in the pulmonary function tests experienced by the oil spill cleanup workers during their initial and later follow-up visits are illustrated in Figure 2. The pulmonary function abnormalities are categorized as normal, mild, moderate, and severe based on their intensity. Of the 44 oil spill cleanup workers, 37 (84%) had normal pulmonary functions at their earlier initial visit after exposure to the oil spill. However, 7 years later after the oil spill, only 18 (48%) cleanup workers had normal pulmonary functions. Mild pulmonary function abnormalities were observed in 4 (9%) of the 44 cleanup workers during their initial visit but these increased to 15 (34%) of the 44 cleanup workers 7 years after their exposure to the oil spill. Similarly, the incidence of moderate pulmonary function abnormality seen initially in 3 (6.8%) workers had increased to 7 (16%) of the 44 cleanup workers from their initial visit to the follow-up visit 7 years after the oil spill. Notably none of the workers (0%) experienced severe pulmonary function abnormalities during their initial visit but 4 (9%) of the 44 workers developed severe pulmonary function abnormalities 7 years after their exposure to the oil spill.

Since oil spill exposure has been shown to affect the cardiac function in birds (32) and fish (33, 34), we examined the cardiac functions in oil spill cleanup workers at their initial visit as well as their follow-up visit at 7 years later. Of the 44 oil spill cleanup workers 23 (52%) experienced some type of cardiac function abnormalities during their initial visit. These abnormalities included abnormal ECG, ventricular conduction delay, anterior fascicular block, sinus rhythm nonspecific T wave, sinus bradycardia ST and T wave abnormality, sinus rhythm early repolarization, and ventricular hypertrophy. These abnormalities persisted even 7 years after the disaster in most of the oil spill cleanup workers (*n* = 18, 41%).

## Discussion

The BP Deepwater Horizon oil spill in the Gulf of Mexico is considered the largest marine oil spill in global history (1). Tens of thousands of individuals participated in the oil spill cleanup activities and were exposed to the various toxic components of the spill (8). Given the toxicological properties of the oil components and the chemicals use to break up the oil slick, exposure to the BP oil spill resulted in health risks for those people who participated in its cleanup operations. Therefore, we have initiated multiple studies to investigate the long-term adverse health consequences of the BP oil spill exposure in workers who participated in the cleanup activities along the Gulf coast (13, 30, 31).

In our previous study, we reported early clinical findings among the workers involved in the oil spill cleanup operation (30). In this follow-up study, we have evaluated the long-term effects of this oil spill exposure among those participants involved in its cleanup activities 7 years after their exposure. To the best of our knowledge, this is the first and only longitudinal study that has evaluated any of the long-term hematological or hepatic adverse health effects of the Gulf oil spill among those subjects involved in its cleanup activities 7 years after the disaster. In addition, this study has evaluated the long-term illness symptoms and complaints, as well as pulmonary and cardiac functions seen in the oil spill exposed cleanup workers (30).

Assessment of hematological markers such as WBC counts, platelet counts, hemoglobin, hematocrit, BUN levels, etc. are routinely used to diagnose a variety of human diseases including malignant tumors. The findings of this longitudinal study performed 7 years after the occurrence of the oil spill disaster shows that subjects who had participated in the cleanup activities had altered profiles of their hematological and hepatic functions when compared with those of the unexposed subjects. More specifically, the oil spill exposed workers had persistently increased mean WBC counts, hemoglobin, hematocrit, APS, AST, and ALT, and reduced platelet counts as well as BUN levels even 7 years after the oil spill disaster. The findings of this study support our previous study findings in which we reported that those workers who were exposed to the oil spill during its cleanup operations had significant hematological and hepatic alterations (30). Another important finding of this long-term study is that most workers have now developed chronic rhinosinusitis (91%) and reactive airway dysfunction syndrome (45%) as new symptoms up to 7 years after their oil spill exposure. These symptoms were not routinely reported during their initial visit which occurred soon after their oil spill exposure. There are no existing studies in literature assessing the long-term human health effects of an oil spill exposure on the hematological, hepatic, pulmonary, or cardiac functions to compare with our study findings.

The long-term effects of the BP oil spill on exposed cleanup workers produced an increased prevalence of illness symptoms such as shortness of breath, headaches, skin rash, chronic cough, weakness, dizzy spells, painful joints, and chest pain 7 years after their exposure to the oil spill. Previously, several studies have likewise reported an incidence of acute somatic symptoms in oil spill exposed or oil polluted subjects (8, 23–26, 35, 36). However, one study by Zock and coinvestigators (28) assessed long-term respiratory symptoms in cleanup workers 5 years after their exposure to the Prestige oil spill. These authors reported that the oil spill exposed workers had persistent respiratory symptoms including shortness of breath, wheeze, cough, and phlegm 5 years after their exposure to the oil spill (28). The findings of the long-term persistent respiratory health effects of the Prestige oil spill exposure support our study findings. The results of our study further reveal that intensive participation even for short periods (only few weeks) in the oil spill cleanup work activities has resulted in persistent long-term adverse health effects. The GuLF STUDY which is a prospective study designed to investigate the relationships between oil spill exposures and multiple potential physical and mental health effects may shed more light on the long-term adverse effects of oil spill exposure in affected populations (37). The Women and Their Children’s Health study has been designed to evaluate the mid-term to long-term physical, mental, and behavioral health effects of the oil spill exposure (8, 38).

In general, an oil spill is composed of a complex mixture of multiple toxic chemicals; its exposure can cause respiratory illnesses and impairment of pulmonary functions (39–41). Therefore, we assessed the pulmonary functions in the oil spill cleanup workers during their initial and later follow-up visits. Our findings indicate that the incidence of deteriorated pulmonary functions had increased over twofold from their initial visit (*n* = 7) to their later follow-up visit (*n* = 18) 7 years after their exposure to the oil spill. The moderate-to-severe pulmonary function abnormalities were more common 7 years after their exposure to the oil spill. Although the precise mechanisms of how the oil spill exposure may have caused impairment of their pulmonary functions are not precisely known, we believe that it is in part due to the exposure of the toxic chemicals found in the crude oil mixture, and their caustic effects on the pulmonary parenchyma during inhalation of the vaporized organic molecules.

Previous studies have shown that oil spill exposure can affect cardiac function in birds (32) and fish (33, 34). Therefore, we evaluated the cardiac function in the oil spill cleanup workers. We found several types of abnormal cardiac functions in those workers involved in the oil spill cleanup operations. These included abnormal ECGs, ventricular conduction delays, anterior fascicular blocks, sinus rhythm nonspecific T waves, sinus bradycardia ST and T waves, sinus rhythm early repolarizations, and ventricular hypertrophy. Given the age of the cleanup workers, we would not expect to see such findings. Over the long term, these cardiac function abnormalities were decreased slightly as compared to the abnormalities seen during their initial visit. We believe that these slight improvements are due to the muscular repair of this organ over time versus the continued damage of the pulmonary tissues and later scarring. Collectively these findings suggest that intensive participation for only several weeks in oil spill cleanup activities resulted in persistent long-term adverse health effects.

The following limitations should be considered when interpreting the study findings. While our study does provide a longitudinal perspective on hematological and hepatic functions in oil spill cleanup workers, it is limited by its use of only initial and the follow-up evaluations and a sample size as a majority of the subjects elected not to return for a follow-up assessment. The limited sample size may have influenced the statistics of the study findings. The existence of methodological challenges particularly studying dynamic human health effects of the oil spill disaster due to the lack of pre-disaster data is another limitation of this study. As is common in disaster research, in this study we did not have pre-disaster health data for all the oil spill cleanup workers and therefore cannot identify all causal factors for the significant alterations found in the hematological, hepatic, pulmonary, and cardiac functions of the oil spill cleanup workers. However, our inclusion of a comparison group that did not participate in the oil spill cleanup operations allowed us to assess relative differences between the oil spill exposed and unexposed groups during their initial and 7-year follow up visits after the disaster. While we recognize that measuring oil spill exposure is difficult due to a lack of validated methodologies and tools of the actual exposure amount that each worker received. We believe it is very unlikely that there was much exposure misclassification of the study participants who were involved in the oil spill cleanup activities.

Despite these study limitations, our findings can clearly demonstrate that exposure to an oil spill is associated with both short-term and long-term adverse health effects in workers involved in its cleanup operations. Specifically, the people who participated in the cleanup activities of the BP oil spill experienced persistent alterations or worsening of their hematological, hepatic, pulmonary and cardiac functions, and prolonged illness symptoms that were still present or worsened 7 years after their exposure. These findings further suggest that workers who are involved in oil spill cleanup operations should be followed consistently over time to detect any long-term toxicities of their oil spill exposure. Since the oil spill contained carcinogenic agents, a latent period exists before these worsening symptoms manifest themselves and progress to malignant diseases. Serial periodic health checkups and routine laboratory blood work as well as high-resolution imaging and pulmonary and cardiac function assessments are necessary to monitor the long-term adverse health effects in workers who participate in oil spill cleanup activities to detect any future malignant transformation.

## Conclusion

The results of this long-term investigation indicate that subjects involved in the BP oil spill cleanup operations experienced persistent alterations in their hematological, hepatic, pulmonary, and cardiac functions. The hematological alterations include increased mean WBC counts, hemoglobin, hematocrit, and reduced platelet counts as well as BUN levels among the oil spill cleanup workers even 7 years after the oil spill disaster. Hepatic alterations included the increased ALP, AST, and ALT levels in the serum indicate hepatic injury in the workers involved in oil spill cleanup operations. A majority of the oil spill cleanup workers developed chronic rhinosinusitis and reactive airway dysfunction syndrome as new symptoms during their later follow-up visit. The incidence of pulmonary function abnormality in the oil spill exposed workers increased substantially from their initial evaluation to their later follow-up assessment. The cardiac abnormalities that were seen in the initial visit persisted even 7 years after the disaster in most of the oil spill cleanup workers. In addition, the oil spill exposed workers reported prolonged or worsening illness symptoms that were present even 7 years after their initial exposure. Additional studies are being conducted to further understand other long-term potential toxic health effects of the BP oil spill exposure among those workers involved in the Gulf oil spill cleanup operations.

## Verification

All authors listed in the manuscript had access to the data and a role in preparing the manuscript.

## Ethics Statement

The study was approved by the University Cancer Diagnostic Centers Institutional Review Board (Quorum Review IRB). As this study involved the retrospective review of medical records of subjects attending the University Diagnostic Centers as part of an organization database, waiver of informed consent was approved by the Quorum Review Board.

## Author Contributions

Both authors conceived and the study design; collected, analyzed, and interpreted the data; and drafted and wrote the manuscript. Both authors read and approved the final manuscript.

## Conflict of Interest Statement

The authors declare that the research was conducted in the absence of any commercial or financial relationships that could be construed as a potential conflict of interest.

## Abbreviations

ALP, alkaline phosphatase*;* ALT, alanine amino transferase; AST, aspartate amino transferase; BUN, blood urea nitrogen; WBCs, white blood cells.
